# In vivo validation of gated myocardial SPECT imaging for quantification of small hearts: comparison with cardiac MRI

**DOI:** 10.1186/s13550-015-0156-5

**Published:** 2016-02-09

**Authors:** Chisato Kondo, Eri Watanabe, Mitsuru Momose, Kenji Fukushima, Koichiro Abe, Nobuhisa Hagiwara, Shuji Sakai

**Affiliations:** Department of Diagnostic Imaging and Nuclear Medicine, Tokyo Women’s Medical University, 8-1, Kawada-cho, Shinjyuku-ku, Tokyo, 162-8666 Japan; Department of Cardiology, Aoyama Hospital, Tokyo Women’s Medical University, Tokyo, Japan; Department of Cardiology, Tokyo Women’s Medical University, Tokyo, Japan

## Abstract

**Background:**

In patients with small hearts, the Quantitative Gated single-photon emission computed tomography (SPECT) (QGS) program frequently underestimates the left ventricular (LV) end-systolic volume (ESV) and overestimates the ejection fraction (EF). A newly developed cardiac software program, cardioREPO/EXINI heart (cREPO), has been proposed to more accurately quantify small hearts using active shape modeling and a volume-dependent edge correction algorithm for LV delineation. The aim of this study was to validate cREPO in vivo for measuring the LV volumes and EF of both small and non-small hearts, in comparison with values obtained via cardiac MRI (CMR).

**Methods:**

We performed stress ^99m^Tc-MIBI SPECT and CMR within a 30-day interval for 44 patients (mean age, 66 years; 27 men). Resting EF, end-diastolic volume (EDV), and ESV with QGS and cREPO were compared with values obtained via CMR.

**Results:**

The subjects consisted of 17 small and 27 non-small hearts. CMR yielded EDV, ESV, and EF values of 135 ± 31 ml (mean ± SD, range 85–217 ml), 57 ± 21 ml (27–105 ml), and 60 ± 6 % (45–70 %), respectively. Compared with CMR, both QGS and cREPO systematically underestimated both EDV and ESV and overestimated EF. The magnitude of the overestimation of EF by QGS, compared with CMR, correlated strongly with the given EF values (*r* = 0.71, *P* < 0.0001). In contrast, no significant correlation was seen with cREPO (*r* = 0.18, *P* = 0.24). In addition, no significant correlation was found between the magnitude of the underestimation of ESV and heart size with cREPO (*r* = 0.03, *P* = 0.83). Thus, cREPO provided a relatively constant 9 % overestimation of EF values relative to CMR, for the studied EF range for both small and non-small hearts.

**Conclusions:**

The use of the new algorithm of cREPO ameliorated exaggerated EF in small hearts but not resolved completely. The program provided a constant 9 % overestimation for both small and non-small hearts, which should be carefully taken into account for clinical assessment of LV function.

## Background

Electrocardiography (ECG)-gated myocardial perfusion single-photon emission computed tomography (SPECT) provides composite information regarding perfusion and function and is widely utilized in the current clinical practice. A number of studies have reported that functional parameters derived from quantification of SPECT, such as ejection fraction (EF) and left ventricular (LV) volumes, correlate well with those obtained from contrast left ventriculography, multi-gated nuclear angiography, and MRI [1, 2]. Such functional parameters have incremental prognostic value over perfusion information [3, 4]. However, it is well known that in subjects with small hearts, LV end-systolic volume (ESV) is underestimated and EF is overestimated [5–7]. The frequency of small LV is greater than 70 % of patients in some populations, such as Japanese women [8]. As many such patients are precluded from receiving an appropriate diagnosis via a uniform, normal threshold [9], this suggests that the algorithm should be corrected for the functional assessments of small LV.

A new cardiac software package, cardioREPO/EXINI heart (cREPO), developed by Exini Diagnostics (Lund, Sweden) in collaboration with FUJIFILM RI Pharma (Tokyo, Japan) and Kanazawa University (Ishikawa, Japan), has been proposed to more accurately quantify small hearts using an active shape LV modeling and volume-dependent edge correction algorithm for LV delineation [10]. This method has been evaluated by using digital phantom experiments, a normal database in Japan, and a clinical series of consecutive patients with small and normal-sized hearts [10]. However, a direct comparison with true LV volumes using a reference method has not been accomplished for the patient data.

The purpose of the current study was to validate cREPO in vivo for measuring LV volumes and EF of both small and non-small hearts, in comparison with values obtained via cardiac MRI (CMR). The characteristics of LV quantification with cREPO were also compared with those produced by the widely used QGS software [11].

## Methods

### Definition of a small heart

As in a previous report [10], in this study, a small heart was defined as a heart with an ESV of ≤20 mL, as calculated using the QGS software.

### Patients

This study was approved by the institutional review board at Tokyo Women’s Medical University (reference number: 140706, approved on 2014/08/29), and written informed consent was obtained from all participants. The study was performed from September 2014 to September 2015. Patients who had been scheduled for clinical myocardial perfusion examinations due to suspected or known coronary artery disease were asked to take part in the study just before undergoing the nuclear tests. A total of 51 patients (33 men, 18 women) agreed to participate at the initial enrollment. For screening of the study candidates, the patients’ medical records were checked regarding gender, body size, and cardiovascular information, so that small and non-small hearts were evenly distributed among the studied patients. Subjects aged <20 years or showing cardiac arrhythmia >5 bpm at rest were excluded. ECG-gated myocardial perfusion SPECT was performed according to a standard stress and rest protocol using bicycle exercise or adenosine infusion. A total dose of 740 MBq of ^99m^Tc-MIBI was used with one third initially administered for stress and the remainder administered for a rest scan 3 to 4 h later.

### SPECT data acquisition and reconstruction

A dual-head SPECT/CT system (BrightView XCT, Philips, Best, The Netherlands) was used for data acquisition and reconstruction. A total of 30 projection data sets were obtained in a 64 × 64 matrix over a 180° arc. The acquisition time was 40 s after stress and 30 s at rest for each projection. Hardware zooming at ×1.46 was applied for both small and non-small hearts. A cardiac cycle was divided into 16 frames. R-R intervals with an average of ±20 % on ECG monitoring were accepted for gating.

The SPECT images were reconstructed via a two-dimensional ordered subset expectation maximization method (iteration 3, subset 10). A Butterworth filter (cutoff frequency 0.58 cycles/cm, order 8) was applied for post-smoothing of the images. Neither CT attenuation correction nor scatter correction was used for gated images. For non-gated data, both images with and without these corrections were generated.

### SPECT data analysis

Gated SPECT data were processed using a standard software package to reconstruct short-axial images. To quantify LV function, we used a standard method using the QGS software (version 2012.1, Cedars Sinai Medical Center, Los Angeles, CA, USA), in which the LV inner and outer surfaces and valve plane were automatically generated on short-axis images. When the fully automatic LV segmentation failed, manual corrections for the basal valve planes were applied using constraints to a specific basal position.

The new method for LV delineation, cREPO, was based on a method which has been previously described in detail [12], in which a heart-shaped LV model and an active shape algorithm are employed. Briefly, after automated location of the LV, the heart-shaped LV model is adjusted in an iterative process to optimize the fit of the mid-myocardial surface to the three-dimensional image data of the first frame, and then to subsequent frames separately without constraints regarding LV basal motion. The endocardial and epicardial surfaces are defined symmetrically on each side of the surfaces as the position corresponding to 75 % of the maximal pixel count along each sampling profile perpendicular to the LV wall [10, 12]. Finally, the LV volume was calculated using the endocardial surface and the valve plane. No manual corrections were applied for LV delineation by cREPO.

**Fig. 1:**
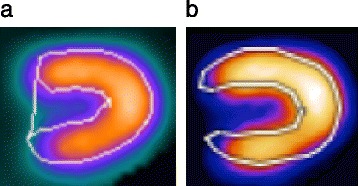
Myocardial boundaries at end systole of a patient with a small heart, detected by QGS **(a)** and cREPO **(b)**

The method was additionally modified and adjusted for small hearts by shifting the endocardial and epicardial surfaces in the epicardial direction prior to LV volume calculations [10] (Fig. 1). The size of the shift was calculated using the non-gated mid-ventricular volume of the LV, using a second degree univariate polynomial equation [10]. The equation provided a shift of 3.5 mm at a mid-ventricular volume of 0 ml, and a decreasing shift up to a volume of 85 ml, for which the shift was 0 mm. For mid-ventricular volumes >85 ml, no adjustment was used [10].

### CMR imaging and analysis

For each patient, CMR was scheduled and accomplished within an interval of <30 days after the SPECT examinations. All imaging was performed on a 1.5T MRI scanner (Achieva, Philips, Best, The Netherlands) with a five-element phased array cardiac coil. Cine steady state free precession sequences were utilized on long-axis, two-chamber, four-chamber, and short-axis views encompassing the whole LV, with no gaps between images. Cine sequences with retrospective cardiac gating were used with the following parameters: cardiac phase =20, TR/TE =3.2/1.6 ms, slice thickness =10 mm, gapless between slices, flip angle =60°, matrix =192 × 256, field of view =380 × 380 mm. All examinations were transferred to a dedicated workstation for subsequent image analysis.

Image analysis was performed manually using QMass MR (version 7.6, Medis, Leiden, The Netherlands) [13] by a CMR expert (EW who has 9 years of experience in CMR analysis) in a totally blind fashion to the SPECT findings. The ES and end-diastolic (ED) frames were visually selected. The basal slice was selected with the aid of long-axis cine view images. Slices at the apex were included when blood was clearly visible. Contours were drawn manually by tracing the endocardial and epicardial borders in every slice at ED and ES. Contour tracing was aided by reviewing the multiple phase scans in the movie mode. Papillary and trabecular muscles were considered to be inside the LV cavity. LV volumes and EF were derived from all short-axis images according to a modified Simpson’s rule.

The same observer who measured all CMR data in duplicates with an interval of >3 months separated from each other determined the intra-observer reproducibility of the analysis. The coefficient of variation (CV) for the repeated measurements was calculated by dividing the SD by the mean of the two values. The root mean square values of these CVs, representing the overall intra-observer variability, for end-diastolic volume (EDV), ESV, and EF were 2.1, 7.8, and 3.6 %, respectively.

### Regional myocardial perfusion and wall motion analysis

**Fig. 2:**
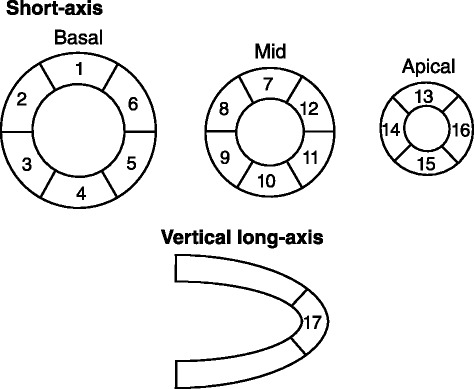
Numbering of 17 myocardial segments

We assessed regional myocardial perfusion using non-gated SPECT at rest and regional wall motion using CMR, QGS, and cREPO, according to 17 segments model [14] (Fig. 2). Myocardial perfusion defects were diagnosed visually using images with and without CT attenuation correction. Wall motion abnormalities were graded visually with the aid of cinematic display of gated images according to five scores (0 =normal motion, 1 =mildly or moderately decreased motion, 2 =severely decreased motion, 3 =akinesis, and 4 =dyskinesis).

### Statistical analysis

All data are expressed as mean ± SD (range), except where otherwise indicated. Differences in the data among CMR, QGS, and cREPO were evaluated using a paired comparison via the Friedman test for repeated measurements of data, and gender-related differences were compared via the Kruskal-Wallis test. Dunn’s multiple comparisons were used for post hoc testing of the analysis of variance. Least squares linear regression and Bland-Altman analyses were used to compare variables between CMR and SPECT. The magnitude of underestimation of volumes by SPECT was compared between EDV and ESV by the Wilcoxon matched-pairs signed rank test. Dispersions of EF differences between QGS minus CMR and cREPO minus CMR were compared using the *F*-test. Agreements of wall motion analysis between CMR and QGS and CMR and cREPO were evaluated via the weighted kappa statistics using quadratic weights [15]. A value of *P* < 0.05 was considered significant.

## Results

### Study subjects

Among the 51 patients initially enrolled in the study, five declined a CMR examination and discontinued participation. In addition, two patients with outlier EDV values of ≥240 ml via CMR were excluded from further evaluations.

The remaining 44 patients (27 men, 17 women; age 66 ± 11 years) had body surface areas of 1.67 ± 0.17 m^2^ (1.32–1.99 m^2^). The diagnosis for these patients consisted of angina pectoris or silent myocardial ischemia (*n* = 20), old myocardial infarction (*n* = 5), coronary artery disease documented by CT coronary angiography (*n* = 3), chronic kidney disease (*n* = 6), ECG abnormality (*n* = 3), and other coronary risk factors (*n* = 7). Nine patients were status after percutaneous coronary intervention and one after coronary bypass grafting. Among these patients, 17 (39 %) and 11 (25 %) had an ESV of ≤20 ml with QGS and cREPO, respectively (*P* = NS, chi-square test).

### Volumes and EF via CMR, QGS, and cREPO

LV volumes and EF via CMR, QGS, and cREPO are summarized in Table 1. Both QGS and cREPO systematically underestimated EDV and ESV and overestimated EF, compared with values from CMR. We analyzed gender-related difference in LV volumes and EF using CMR, QGS, and cREPO (Table 2). None of these variables by CMR showed significant gender-related difference. ESV and EF using QGS significantly differed between men and women, but EF using cREPO did not. EDV determined by QGS and LV volumes by cREPO showed tendency of the difference, but not reaching statistical significance.

**Table 1:** Paired comparisons between variables determined by CMR, QGS, and cREPO

Variable	CMR	QGS	cREPO	Statistics (*P* < 0.05)
EDV (mL)	135 ± 31 (85–217)	80 ± 26 (42–150)	93 ± 26 (50–152)	CMR vs QGS, CMR vs cREPO, QGS vs cREPO
ESV (mL)	57 ± 21 (27–105)	30 ± 19 (8–85)	29 ± 12 (12–69)	CMR vs QGS, CMR vs cREPO
EF (%)	60 ± 6 (45–70)	66 ± 12 (37–86)	69 ± 12 (55–77)	CMR vs QGS, CMR vs cREPO, QGS vs cREPO

Parenthesis indicates range of each value

**Table 2:** Variables determined by CMR, QGS, and cREPO in men and women

Variable	CMR			QGS			cREPO		
	Men (*n* = 27)	Women (*n* = 17)	*P* value	Men (*n* = 27)	Women (*n* = 17)	*P* value	Men (*n* = 27)	Women (*n* = 17)	*P* value
EDV (mL)	145 ± 33	118 ± 19	>0.99	90 ± 26	63 ± 15	0.094	104 ± 26	77 ± 16	0.082
ESV (mL)	62 ± 21	47 ± 17	>0.99	37 ± 19	18 ± 9	0.005	34 ± 12	21 ± 5	0.086
EF (%)	58 ± 7	63 ± 4	>0.99	61 ± 11	73 ± 9	0.0006	67 ± 5	72 ± 5	0.63

**Fig. 3:**
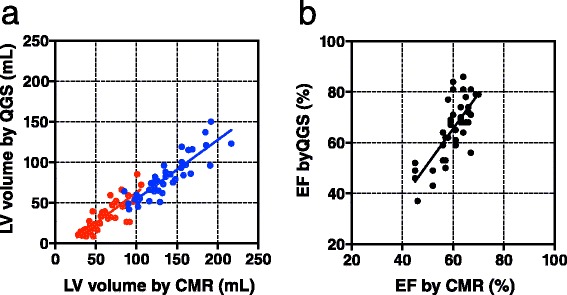
Linear regression plots between QGS and CMR of LV volumes **(a)** (*blue circles* indicate EDV; *red* indicates ESV) and EF (**b**)

**Fig. 4:**
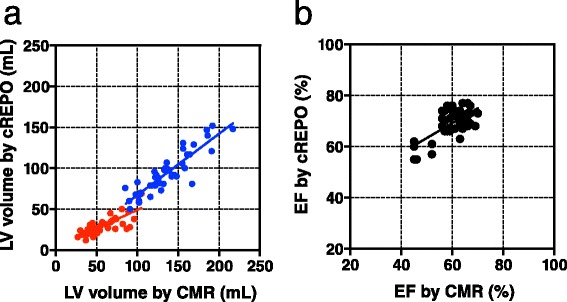
Linear regression plots between cREPO and CMR of LV volumes **(a)** and EF **(b)** *Blue* and *red circles* indicate as the same as Fig. 3

The overall correlation between QGS and CMR measurements for LV volumes was good (EDV *r* = 0.94, SEE =12 ml, *P* < 0.0001; ESV *r* = 0.84, SEE =10 ml, *P* < 0.0001; EF *r* = 0.75, SEE =7.8 %, *P* < 0.0001) (Fig. 3) and was also good between cREPO and CMR values for volumes (EDV *r* = 0.90, SEE =12 ml, *P* < 0.0001; ESV: *r* = 0.80, SEE =7 ml, *P* < 0.0001; EF *r* = 0.66, SEE =4.2 %, *P* < 0.0001) (Fig. 4).

**Fig. 5:**
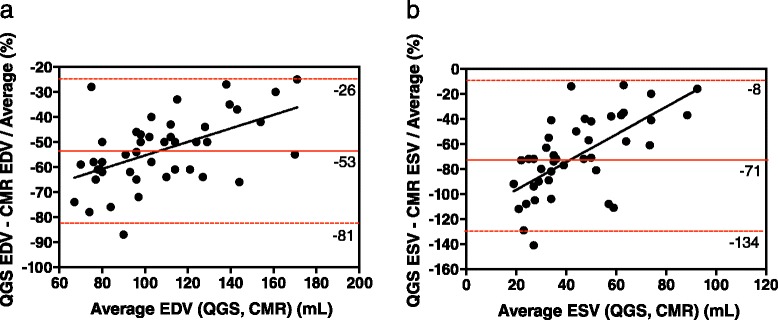
Bland-Altman plots of LV volumes measured by QGS and CMR for EDV **(a)** and ESV **(b)**. *Horizontal red lines* indicate mean ± 1.96 SD

**Fig. 6:**
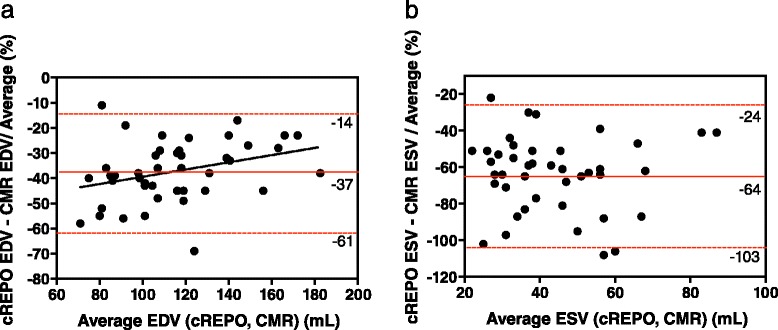
Bland-Altman plots of LV volumes measured by cREPO and CMR for EDV **(a)** and ESV **(b)**. *Horizontal red lines* indicate as the same as Fig. 5

For Bland-Altman analysis of LV volumes, the magnitude of the underestimation of volumes was expressed as the percent difference of two measurements divided by the average. Bland-Altman plots showed that the systematic and random differences (mean ± 1.96 SD) between the QGS and CMR measurements of EDV and ESV were −53 ± 28 and −71 ± 63 %, respectively (Fig. 5). The corresponding differences for the cREPO and CMR measurements of EDV and ESV were −37 ± 24 and −64 ± 40 %, respectively (Fig. 6). The magnitude of the underestimation of LV volumes of QGS and cREPO, as compared with CMR, significantly correlated with heart size except for ESV with cREPO (Figs. 5 and 6) (EDV with QGS and CMR *r* = 0.53, *P* = 0.0002; ESV with QGS and CMR *r* = 0.65, *P* < 0.0001; EDV with cREPO and CMR *r* = 0.33, *P* = 0.03; ESV with cREPO and CMR *r* = 0.03, *P* = 0.83). The magnitude of the underestimation of ESV was significantly larger than that for EDV for both QGS and cREPO (both *P* < 0.0001).

**Fig. 7:**
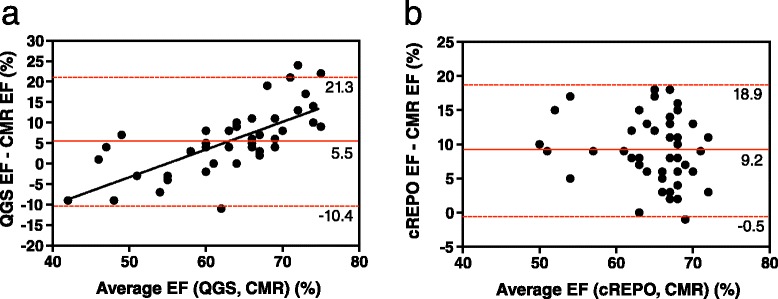
Bland-Altman plots of EFs measured by QGS and CMR **(a)** and cREPO and CMR **(b)**. *Horizontal red lines* indicate as the same as Fig. 5

**Fig. 8:**
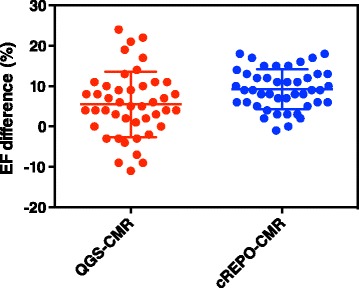
Systematic and random differences of EF between QGS and CMR (*red circles*) and cREPO and CMR (*blue circles*). *Horizontal lines* indicate mean ± 1 SD

Bland-Altman plots of EF indicated that the systematic and random differences (mean ± 1.96 SD) of EF between QGS and CMR and cREPO and CMR were 5.5 ± 15.8 and 9.2 ± 9.7 %, respectively (Fig. 7). The magnitude of overestimation of EF by QGS, as compared with CMR values correlated strongly with the given EF values (*r* = 0.71, *P* < 0.0001); however, no significant corresponding correlation was seen with cREPO (*r* = 0.18, *P* = 0.24) (Fig. 7). The dispersion of the differences in EF between cREPO and CMR was significantly less than that of between QGS and CMR (variance ratio = 2.68, *P* = 0.002) (Fig. 8).

### Regional wall motion and myocardial perfusion

Comparisons of 17 segment-based, wall motion scores for the studied patients between CMR and QGS and CMR and cREPO are shown in Tables 3 and 4. The weighted kappa values between CMR and QGS and CMR and cREPO were 0.713 and 0.574, respectively, indicating good or moderate agreement of the wall motion scores between these techniques. In five patients with myocardial infarction, locations of perfusion defect on SPECT and abnormal wall motion on CMR, QGS, and cREPO were corresponded well with each other (Table 5). Wall motion scores in the five patients with infarction via CMR, QGS, and cREPO were 0.97 ± 1.40, 0.62 ± 0.88, and 0.44 ± 0.74, respectively (CMR vs. QGS, *P* = NS; CMR vs. cREPO, *P* < 0.05; QGS vs. cREPO, *P* = NS), suggesting cREPO underestimates wall motion abnormality in comparison with CMR.

**Table 3:** Comparison of wall motion scores between CMR and QGS

	CMR					
QGS	0	1	2	3	4	
0	694	13	1	0	0	708 (94.7 %)
1	12	5	1	2	2	22 (2.9 %)
2	2	2	3	7	3	17 (2.3 %)
3	0	0	0	1	0	1 (0.1 %)
4	0	0	0	0	0	0 (0.0 %)
	708 (94.7 %)	20 (2.7 %)	5 (0.7 %)	10 (1.3 %)	5 (0.7 %)	748

Wall motion scores are defined as 0 =normal, 1 =mildly or moderately reduced, 2 =severely reduced, 3 =akinesis, and 4 =dyskinesis

**Table 4:** Comparison of wall motion scores between CMR and cREPO

	CMR					
cREPO	0	1	2	3	4	
0	705	18	3	2	0	728 (94.7 %)
1	2	1	1	3	3	10 (1.3 %)
2	1	1	1	5	2	10 (1.3 %)
3	0	0	0	0	0	0 (0.0 %)
4	0	0	0	0	0	0 (0.0 %)
	708 (94.7 %)	20 (2.7 %)	5 (0.7 %)	10 (1.3 %)	5 (0.7 %)	748

Numeric classification on values are defined as the same as Table 3

**Table 5:** Location of perfusion defect and wall motion abnormality in patients with old myocardial infarction

Case	Diagnosis	Perfusion defect	Wall motion abnormality		
			CMR	QGS	cREPO
1	OMI (I)	3, 4, 5, 10	4, 10	4, 9, 10	4, 10
2	OMI (I)	3, 4, 5, 10, 11, 15	3, 4, 5, 9, 10, 11, 15	3, 4, 5, 9, 10, 11, 15	3, 4, 9, 10, 15
3	OMI (A)	Normal	Normal	Normal	Normal
4	OMI (A)	2, 3, 7, 8, 9, 13, 14, 17	1, 2, 3, 7, 8, 9, 13, 14, 17	2, 3, 7, 8, 9, 13, 14, 15, 17	2, 3, 7, 8, 9, 13, 14, 15, 17
5	OMI (L, I)	1, 4, 5, 7, 10	1, 2, 4, 5, 7, 8, 10, 11	1, 3, 4, 5, 9, 10, 11	3, 4, 9, 10

Numbers indicate location of myocardial segment as shown in Fig. 2

*A* anterior, *I* inferior, *L* lateral, *OMI* old myocardial infarction

## Discussion

In the present study, the combination of active shape modeling and a volume-dependent edge correction algorithm effectively reduced the volume-dependent effects on ESV and EF of a small heart, which was validated in vivo in comparison with CMR measurements of small and non-small hearts. We found that cREPO provided a constant 9 % overestimation of EF in small and non-small hearts, and, in contrast to QGS, EF determined by cREPO did not differ between men and women, which is consistent with a previous study [10].

We found a systematic underestimation of EDV and ESV by QGS and cREPO compared to CMR, which is consistent with previous studies comparing gated SPECT and CMR [16–18]. There could be several underlying mechanisms why gated SPECT underestimates LV volumes. CMR has higher spatial resolution compared to SPECT, which affects delineation of the endocardial border resulting in larger endocardial volumes with CMR. According to general practice of CMR, epicardial and endocardial borders in the ED and ES phase of the cardiac cycle are manually drawn for LV quantification. Here, the manual tracing of the endocardial borders usually includes the papillary and trabecular muscles in the blood volume [13]. In cardiac SPECT, however, trabecular and papillary muscles are avid to perfusion tracers and can be inevitably excluded from the blood volume. CMR allows inclusion of the membranous part of the ventricular septum, the atrioventricular valve plane, and LV outflow tract as a part of LV cavity, which are not part of LV volume acquisition with gated SPECT [17, 18]. Taken together, these factors may contribute to underestimation of the basal LV volume with gated SPECT.

This study indicated that the use of the new algorithm in cREPO was effective in resolving the volume-dependent progressive underestimation of ESV associated with a small heart. The volume-dependent progressive underestimation of volumes is observed not only for a chamber ≤20 ml but is also observed to occur as the volume decreased below 100 ml in a simulation study [5]. This volume-dependent effect is affected by various factors including system resolution, reconstruction filter, and hardware zooming [5], all of which affects volume measurements more seriously as heart size decreases [5]. The software algorithm utilized for edge detection may also affect the measurements. However, the overestimation problem of EF in a small heart is commonly observed not only with QGS but also with both the Emory Cardiac Toolbox [19] and 4DM-SPECT [20] when assessing patient data [21]. Thus, the volume-dependent algorithm for LV border detection may represent the first such success for small hearts in vivo relative to commercially available software, as shown in this study.

The volume-dependent edge correction algorithm was only applied for hearts with a mean volume of ≤85 ml. However, in this study, a volume-dependent underestimation of EDV was observed not only in QGS but also in cREPO, suggesting the correction may also be required for a heart with a volume of >85 ml. We also noted that all of the four study patients with mildly to moderately depressed EF values of ≤46 % via CMR showed EF values of ≥55 % with cREPO. The same finding, namely the overestimation of mildly depressed EF with cREPO, was also found in a previous study in the literature [10]. These findings suggest that the overestimation of EF is consistently observed in patients with mildly depressed LV function, and, thus, a patient with preserved but borderline EF by cREPO should be carefully taken into account of possible LV dysfunction in a clinical setting.

Some limitations must be considered in this study. First of all, this study did not include hearts with severe LV dysfunction. We found two patients with severely depressed EF (28 and 29 % with CMR and 24 and 38 % with cREPO) during the study period; however, as their volume data were outliers of the studied subjects, these patients were excluded from the final analysis. Secondly, this study suggests that cREPO underestimates the severity of wall motion abnormality at infarcted area. This finding may partly explain the overestimation of EF by cREPO in patients with mildly depressed LV function in this study, but the number of the patients with myocardial infarction was very limited. To clarify the accuracy of cREPO assessing global and regional LV dysfunction, further studies may be required, including substantial number of patients with myocardial infarction and various degrees of LV dysfunction. Thirdly, we employed 20 phases per cardiac cycle for CMR acquisition, leading to temporal resolution of the studied subjects with 47 ± 10 ms (range, 35–64 ms). According to a recent standardized CMR protocol, temporal resolution for CMR is recommended as ≤45 ms [22]. In one study, there was no significant influence of temporal resolution of 45–90 ms on EDV, compared with a standard of reference (temporal resolution of 21 ms) [23]. They also showed that results were not significant in a comparison of EF at temporal resolution of 45 and 21 ms, but significant at 60 ms by 3.4 % decrease compared with the value at 21 ms. We found 34 patients (11 small heart and 23 non-small heart) with temporal resolution of ≥45 ms and 5 (1 small heart and 4 non-small heart) of ≥60 ms in our study, which may introduce some bias on EF measurements via CMR. However, the number of subjects with the limited temporal resolution was distributed to both small and non-small heart groups. In addition, gated SPECT acquired with 16 frames per cardiac cycle was further limited in temporal resolution compared with CMR, suggesting that bias on EF measurements by limited temporal resolution seems to affect similarly on both groups with heart size, and on both CMR and SPECT measurements. Thus, it is unlikely that limited temporal resolution in our study significantly affects on final conclusions.

## Conclusions

The use of the new algorithm of cREPO ameliorated exaggerated EF in small hearts, but not resolved completely. The program provided a constant 9 % overestimation for both small and non-small hearts, which should be carefully taken into account for clinical assessment of LV function.

## Abbreviations

CMR: cardiac MRI
cREPO: cardioREPO/EXINI heart
ECG: electrocardiography
EDV: end-diastolic volume
EF: ejection fraction
ESV: end-systolic volume
LV: left ventricular
QGS: Quantitative Gated SPECT
SEE: standard error of estimate
SPECT: single-photon emission computed tomography
